# Evaluation of Minimal Residual Disease in Acute Myeloid Leukemia with NPM1 Marker

**Published:** 2016-07-01

**Authors:** Nasrin Alizad Ghandforoush, Bahram Chahardouli, Shahrbano Rostami, Habibeh Ghadimi, Ali Ghasemi, Kamran Alimoghaddam, Ardeshir Ghavamzadeh, Fatemeh Nadali

**Affiliations:** 1 MSc, Department of Hematology, School of Allied Medical Sciences, Tehran University of Medical Sciences, Tehran, Iran; 2 Assistant Professor, Hematology-Oncology and Stem Cell Transplantation Research Center, Tehran University of Medical Sciences, Tehran, Iran; 3 MSc Student, Hematology-Oncology and Stem Cell Transplantation Research Center, Tehran University of Medical Sciences, Tehran, Iran; 4 PhD Student of Hematology, Blood Transfusion Research Center, High Institute for Research and Education in Transfusion Medicine, Tehran, Iran; 5 Professor, Hematology-Oncology and Stem Cell Transplantation Research Center, Tehran University of Medical Sciences, Tehran, Iran; 6 Associate Professor, Department of Hematology, School of Allied Medical Sciences, Tehran University of Medical Sciences, Tehran, Iran

**Keywords:** Acute myeloid leukemia, MRD, NPM1 mutation, Q-RT-PCR

## Abstract

**Background:** Minimal residual disease (MRD) tests provide early identification of hematologic relapse and timely management of acute myeloid leukemia (AML) patients. Approximately, 50% of AML patients do not have clonal chromosomal aberrations and categorize as a cytogenetically normal acute myeloid leukemia (CN-AML). About 60% of adult CN-AML has a mutation in exon 12 of NPM1 gene. This mutation is specific for malignant clone and potentially is a good marker of MRD. In this retrospective study, we set up a quantitative test for quantifying NPM1 type A mutation and AML patients carrying this mutation at the time of diagnosis, were followed-up.

**Materials and Methods** **:** We prepared plasmids containing a cDNA fragment of NPM1 and ABL genes by PCR cloning. The plasmids were used to construct standard curves. Eleven patients were analyzed using established method. Serial PB and/or BM samples (n=71) were taken in 1-3 months intervals (mean 1.5-month intervals) and median follow-up duration after chemotherapy was 11 months (5-28.5 months).

**Results:** In this study, we developed RNA-based RQ-PCR to quantitation of NPM1 mutation A with sensitivities of 10^(-5)^. The percent of NPMmut/ABL level showed a range between 132 and 757 with median of 383.5 in samples at diagnosis. The median NPMmut transcript level log reduction was 3 logs. Relapse occurred in 54.5% of patients (n=6), all cases at diagnosis demonstrated the same mutation at relapse. In patients who experienced relapse, log reduction levels of NPM1 mRNA transcript after therapy were 4 (n=2), 3 (n=2) and 1 log (n=2). Totally, NPMmut level showed less than 5 log reduction in all of them, whereas this reduction was 5-6 logs in other patients.

**Conclusion**: Despite the limitations of this study in terms of sample size and duration of follow-up, it showed the accuracy of set up for detection of mutation and this marker has worth for following-up at different stages of disease. Because of high frequency, stability, specificity to abnormal clone and high sensitivity, NPM1 is a suitable marker for monitoring of NPMc+ AML patients.

Nasrin Alizad Ghandforoush and Bahram Chahardouli equally contributed in this work.

## Introduction

 Acute myeloid leukemia describes by abnormal proliferation of hematopoietic precursors and disturbing the normal hematopoiesis and it is the clinically, cytogenetically and molecularly heterogeneous disorder.   ^1^^,^^2^  AML patients are categorized in three risk groups (favorable, intermediate, or unfavorable) based upon cytogenetic risk factors.^3^

The remarkably heterogeneous nature of cytogenetically normal acute myeloid leukemia (CN-AML) has become more apparent by discovery of multiple molecular lesions in this group of patients that results in difference in response to chemotherapy and clinical outcome.^   3 ^

NPM1 mutations, the most common genetic abnormality has been detected in adult de novo AML, are observed in about 30% of AMLs and about 50%-60% CN-AML. ^1^^,^^3^  The NPM1 gene is located on long arm of chromosome 5 at band q35.^   4 ^ NPM1 wild type is a Nucleocytoplasmic trafficking protein, with localization in the nucleolus.^   1 ^ The important functions of NPM1 are: transport of ribosomal components into the cytoplasm, control of centrosome duplication, reaction with p53, and regulation of ARF-Hdm2/Mdm2-P^53^ oncogenic suppressor pathway. ^1^^,^^5^ 

The NPM1 mutations occur in exon 12 and the most common of these mutations, type A, are as result of a tetra-nucleotide insertion (TCTG) at position 956-959 of NPM1 gene that observe in approximately 80% of cases NPMc_+_ AML. ^1^^,^^6^  Molecular markers are useful in dissection of this heterogeneous group of AML patients into prognostically different subgroups.^   7 ^ This mutation is specific for malignant clone and potentially is a good marker of MRD.^   7 ^

Achieving hematologic complete remission is the first goal in treatment of AML patients.^   8 ^ The majority of AML patients relapse within 3-5 years after diagnosis.^   8 ^ Minimal residual disease (MRD) tests provide early identification of hematologic relapse and timely management of acute myeloid leukemia (AML) patients.^   2 ^ Quantitative RT-PCR method is the most commonly used method for detecting MRD with high sensitivity (1×10^-5^).^   2 ^

Approximately, 50% of AML patients do not have clonal chromosomal aberrations and categorize as a cytogenetically normal acute myeloid leukemia (CN-AML)^   8 ^, whereas FLT3/ITD mutations present in 30-40% of patients with CN–AML. The role of FLT3/ITD mutations as a MRD marker is controversial because it is considered as a relatively unstable marker that may lost at relapse.^   2 ^ The high level of WT1 expression in BM and PB is associated with the presence of disease in AML patients and increase in WT1 levels before hematological relapse can be showing its role in MRD monitoring.^   2 ^ But, WT1 expression in normal PB and BM cells leads to low sensitivity of MRD analysis in AML patients.^   9 ^

NPM1 mutation is more specific, sensitive and stable molecular marker in comparison with the above mentioned markers.^   7 ^ After induction therapy, complete remission rate in AML with normal karyotype (AML-NK) patients with NPM1 mutant was higher than that in AML-NK patients without NPM1 mutant.^   1 ^ NPM1 mutations independently predicts markers for response of chemotherapy because higher complete remission rate achieve only in the NPM1^+^- FLT3^-^ group,^   1 ^ while AML patients with both mutations show the lowest response rate.^   1 ^ Assessment of minimal residual disease predicts early recurrence and long-term survival. ^10^^,^^11^  Molecular assessment of NPM1 mutant transcript levels at two different check points; the double induction therapy and complete consolidation therapy, has equal and important impact on the prognosis. ^10^^,^^11^ 

This study was performed in two steps. First, we set up a quantitative test for quantifying NPM1 type A mutation and in the second step, AML patients carrying this mutation at the time of diagnosis, were followed-up.

## MATERIALS AND METHODS

 Fresh BM and/or PB samples from AML patients with NPMmut A at diagnosis and different time points were collected during follow-up (Ethical code: IR.TUMS.REC.1394.1296). Demographic and clinical characteristics of enrolled patients are summarized in Table 1. Mononuclear cells were separated by ficoll gradient density. Total RNA was extracted with TriPure reagent (Roche) and then, cDNA was synthesized using Thermo Scientific Kit according to the manufacturer‘s instructions. cDNA containing NPM1 A mutation was run as a positive control and used in cloning process to set up NPM1 quantitative test.

**PCR**

Exon 12 type A mutation of NPM1 gene from newly diagnosed sample was amplified by using specific primers. Also, part of ABL gene (as an internal housekeeping gene) was amplified by using normal cDNA sample and specific primers. Primer sequences are given in Table 2.

The 20 μl reaction mixtures contained 10 μl of PCR master mix Ampliqon Master mix Red (Ampliqon, Copenhagen, Denmark), 0.5 µM of each primer and 2 µl of cDNA.

**Table 1:** Demographic and clinical characteristics of the enrolled patients

**No of patients**	11	
**Ages (years)**	Median: 42	Range: 28-63
**Sex**	Male: 6	Female: 5
**AML history**	Denovo AML	-
**FAB** **M5** **M4** **M2** **M1**	4 4 2 1	
**WBC count, × 10** ^9^ **/L**	Median: 30100	Range: 4600-110000
**Bone marrow blasts, %**	Median: 40	Range: 20-94
**Response** **Complete Response Relapse**	11 6	
**Transplantation** **Autologous** **Allogeneic stem cell transplantation**	1 6	
**Follow-up after chemotherapy month**	Median: 11	Range: 5-28.5

**Table 2:** Primer sequences for NPM1mut and ABL Cloning

**Primer**	**Sequences**
NPM1 Cloning-F	GGTCTTAAGGTTGAAGTGTGGT
NPM1 Cloning-R	TCAACTGTTACAGAAATGAAATAAGACG
ABL Cloning-F	CCTTCAGCGGCCAGTAGC
ABL Cloning-R	GGACACAGGCCCATGGTAC

**F:** Forward, **R: **Reverse

Reactions were carried out in ABI verity thermo cycler under temperature conditions consisted of 94^°^C for 4 min, followed by 35 cycles of 95^°^C for 30 sec, 64^°^C for 30 sec, 72°C for 30 sec and ended with 1 cycle72^°^C for 7 min. The PCR products were electrophoresed in 2% (w/v) agarose gel.

**Cloning and screening of recombinant plasmids**

The PCR products were extracted from agarose gels with the YTA Gel Extraction Kit according to the manufacturer's protocol. The PCR products obtained were cloned into the T/A cloning vector (Thermo Scientific kit). The transformed bacteria were cultured on LB agar plate including 20 mg/ml X-GAL, 0.024 mg/ml IPTG and 50 mg/ml ampicillin at 37°C for 24h.

The white colonies were selected and then colony PCR was performed with specific primers for detection of real recombinant colony. The PCR products were confirmed by sequencing. recombinant plasmids were isolated using alkaline lysis method.^   12 ^ For this purpose, the colony was inoculated in to 3-5 ml of LB broth and incubated at 37^°^C over night and in next step, for large scale isolation of plasmid DNA, 200 µl of it was added to 200 ml of freshly prepared LB broth containing ampicillin and then, grown with vigorous shaking for 12-16 hours. Plasmids were extracted according to protocol. Recombinant plasmids were linearized with Hind III (Cinagen) endonuclease and plasmid concentration was measured by spectrophotometer. The number of plasmid copies was calculated with respect to molecular weight of recombinant plasmid. Serial dilutions were prepared and used to create the standard curves of NPM1 and ABL with 5-fold (10^2^-10^6^ copies/5 μl) and 4-fold (10^3^-10^6^ copies/5 μl) dilutions, respectively. The sensitivity of RQ-PCR approach was determined by serial dilutions of cDNA from the cells of a patient with NPM1mut in cDNA from cells of normal healthy donor.

**Real-time-PCR**

Real-time quantitative was carried out using ABI Step One Plus PCR System. The PCR reactions were carried out in a total volume of 20 µl containing 10 µl Taq Man Master Mix (TaKaRa), 0.5 µM of each primer, 0.2 mmol/L of probe and 5 mL of cDNA.

Forward and reverse primers were designed for specific detection of NPM1mut A according to Gorello P et al.^13^ shown in Table 3. ABL gene was used as control gene for normalization of qPCR. A sample from NPM1mut A patient was used as positive control and normal sample (without NPM1 mutation A) was used as a negative control.

Real-time PCR temperature conditions were as follows: initial denaturation 95°C for 30 sec (1 cycle), annealing and exponential phase 95°C for 30 sec, 50°C for 20 sec, 65°C for 20 sec (45 cycle). The results were reported according to NPM1mut/ABL percentage.

## Results

**Figure 1:**
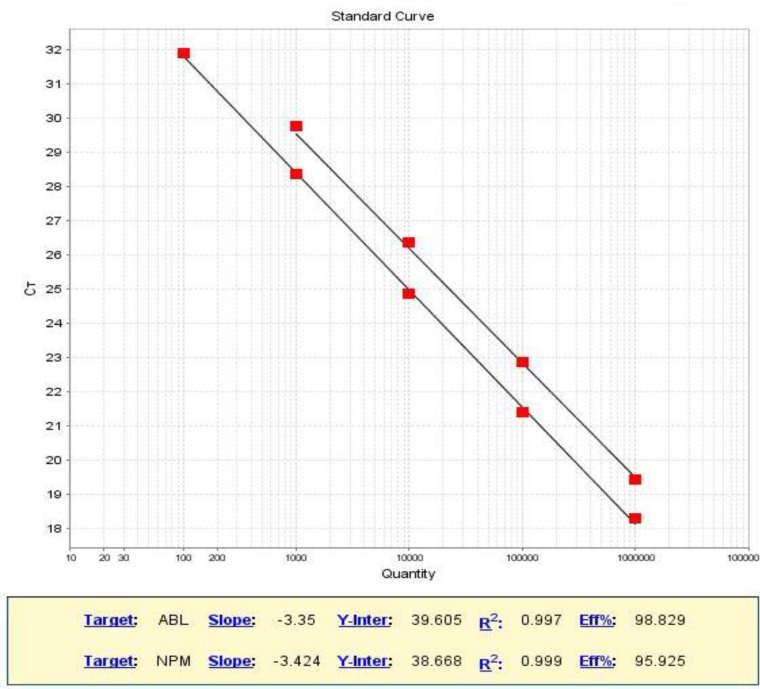
Standard curves are constructed for NPM1mut and ABL genes

 Standard curves were constructed using 5 (10^2^-10^6^ copies/5 µl) and 4-fold (10^3^-10^6^ copies/5 µl) serial dilutions of standard plasmids containing NPM1mut and ABL genes respectively, Figure 1.

**Table 3:** sequences of primers and probs used for quantitative Real-time PCR

**Primers ** **and Probs**	**Sequences**
NPM1 Forward	GAAGAATTGCTTCCGGATGACT
NPM1 Probe	FAM-ACCAAGAGGCTATTCAA-TAMRA
NPM 1mut A- Reverse	CTTCCTCCACTGCCAGACAGA
ABL Forward	TGGAGATAACACTCTAAGCATAACTAAAGG
ABL Probe	FAM-CCATTTTTGGTTTGGGCTTCACACCATT-TAMRA
ABL Reverse	GATGTAGTTGCTTGGGACCCA

The standard curve showed an efficiency of 98.82% based on the slope of -3.35 for ABL gene and an efficiency of 95.92 % corresponds to a slope of -3.42 for NPM1mut gene. The result of sensitivity assay showed that mutant signals could be detected at a ratio of 1:100,000 (sensitivity of 10^-5^). The specificity of method was determined using a sample without mutation that was verified by sequencing and also normal sample was used in each run and no amplification was detected. Eleven AML patients with NPM1mut A were analyzed using established method.

Serial BM or/PB samples (n=71) were taken in 1-3 months intervals (mean 1.5 month intervals) and median follow-up duration after chemotherapy was 11 months (5-28.5 months). Samples were assayed by RNA-based RQ-PCR. NPMmut transcript levels were expressed as percentage ratio of NPMmut to ABL transcripts.

The percent of NPMmut/ABL level showed a range between 132 and 757 with a median of 383.5 in samples at diagnosis. The median NPMmut transcript level log reduction was 3 logs. Relapse occurred in 54.5% of patients (n=6). All cases at diagnosis demonstrated the same mutation at relapse. On the basis of MRD kinetics in 11 patients who were monitored after treatment, distinct patterns of NPM1mut transcript level reduction were observed as follows:

1- Two patients showed good response to treatment with more than 5-log reduction in the NPM1mut levels. They were subjected to allo and auto-HSCT 3 and 4 months after diagnosis and in their first CR, respectively. They did not relapse during the follow-up (mean, 10.5 months).

2- Four patients showed 4-log reduction in the NPM1mut levels. During follow-up, 2 of them had a gradual increase in mutant transcript levels and finally in both of them relapse occurred after 6 months. The 2 other patients showed steady decline in NPM1mut levels (one of them was undergoing allogenic transplantation) and were in complete remission at last follow-up (mean: 20.7 months).

3- Two patients with 3-log reduction in mutant transcripts experienced relapse during average 9.5 months of follow-up. In one case recurrence and death occurred 2 and 3 months after allo-HSCT, respectively.

The other patient was old and after clinical relapse he was treated in clinical trial by low dose cytosar subcutaneous plus arsenic trioxide. At last follow-up (n=12), the patient was still alive.

4- Relapse and death occurred in 2 of the 3 patients with a 1-log reduction in NPM1mut levels (mean follow-up: 7.25 months). The third patient in this group received allo-HSCT. Complete remission and gradual reduction in mutant transcripts were detected during follow-up (8 months).

## Discussion

 Minimal residual disease (MRD) tests provide early identification of hematologic relapse and timely management of acute myeloid leukemia (AML) patients.^   2 ^ Approximately, 50% of AML patients do not have clonal chromosomal aberrations and categorize as a cytogenetically normal acute myeloid leukemia (CN-AML).^        10 ^

Molecular markers are useful in dissection of this heterogeneous group of AML patients into prognostically different subgroups.^   7 ^ About 60% of adult CN-AML have a mutation in exon 12 of NPM1 gene.^   1 ^ This mutation is specific for malignant clone and potentially is a good marker of MRD more than other markers i.e. WT1 and FLT3-ITD.^   3 ^ In this retrospective study, we set up a NPM1 quantitative test and then, AML patients carrying NPM1mut were followed-up for MRD monitoring.

In this study, we developed RNA-based RQ-PCR to quantitation of NPM1 mutation A. Higher sensitivity of RNA previously was shown by Gorello P et al.^        13 ^ Reverse and forward specific primers were used for amplification of 12 exon in NPM1 gene carrying mutation A and probe labeled with fluorescent dyes (FAM and TAMRA), located on exon junction, was used for amplification of cDNA only.^13^ The results showed that monitoring of NPM1 mutation A could be detected with sensitivity of 10^-5^.

This sensitivity is suitable for MRD detection. The sensitivity depends on types of samples (PB, BM) and probes and it can vary between 10^-4^ and 10^-6^.  ^10^^,^^13^ 

In this study, relapse occurred in 54.5 % of patients (n=6). All cases at diagnosis demonstrated the same mutation at relapse. Schnittger S et al. showed that the type of NPM1 mutation was same in 84 paired diagnosis-relapse samples,^        10 ^ and concluded that this marker is stable in different phase of disease. Nevertheless, Kronke J et al. showed that NPMmut level was not detectable in 5 of 45 patients at time of relapse.^        14 ^ The recent study results on a large cohort confirmed previous results as they found that NPM1 mutation was lost at relapse in 5/53 paired PB and/BM samples.^        15 ^

Variable NPMmut level with 3 logs or more differences at diagnosis was reported by Schnittger S et al.^        10 ^ whereas in our study, the level of expression of NPMmut transcript at diagnosis showed less than 1 log difference that was not significant that could be due to small samples size in this study.

In early study by Schnittger, the relationship between level of gene fusions at time of diagnosis and response to treatment was assessed and showed that primary high level of gene fusions was associated with an adverse outcome in patients.

But in their most comprehensive study on patients with NPM1 mutation, they reported that NPMmut level at diagnosis had no significant effect on any of the survival parameters. ^10^^,^^16^ 

In the present study, no significant correlation was found between mutant transcript level and WBC and Plt count at diagnosis. Also, there was no significant association between the initial level of NPMmut and relapse. Due to small sample size of study, more samples are required to approve the conclusion. Barragan E et al. assessed MRD by WT1 and NPM1 markers in 24 NPM1 type A-mutated AML patients, simultaneously. There were 6 relapse samples of 5 patients and the levels of both markers at relapse were higher than at diagnosis in PB and BM samples.^        17 ^ Their results were in accordance with observations made by Schnittger S et al. who indicated that NPM1mut levels at relapse were 2 logs higher than at diagnosis in 84 paired samples of at diagnosis/relapse.^        10 ^

In present study, in 1 of 6 patients with relapse, NPM1mut/ABL% level at first relapse was similar to at diagnosis, which was increased to 1.5 logs at second relapse. Also, the mutant transcript level at relapse increased to about 1 log in 2 patients but in 3 latter cases it was lower than at diagnosis. These results could be related to collection of samples at time of molecular relapse and before clinical relapse.

In a study conducted by Schnittger S et al. different patterns of treatment response were defined in monitoring of MRD after first-line treatment and it was reported that patients who had at least a 5-log reduction in NPM1mut levels showed good response to treatment.^        10 ^ In the present study, 6 patients experienced recurrence whose NPM1mut reduction levels were less than 5 logs after treatment. The patient with ITD^−^- NPM1^+^ displayed less aggressive disease compared with FLT3/internal tandem duplication (ITD)^+^ patient in third group. The latter case did not respond to treatment and died after recurrence. FLT3/ITD mutation can trigger anti-apoptotic and cell proliferation pathways, particularly through STAT 5 that may contribute to the leukemic phenotype.^   1 ^ This may show that FLT3-ITD statue should be considered to interpret results of NPM1 statue the point that is mentioned in Falini B et al.’s studies.^1^

## CONCLUSION

 Totally, despite limitations of the current research, in terms of samples size and duration of follow-up, this study clearly demonstrates the accuracy of test method and shows that the levels of mutant NPM1 reduction after induction treatment can be important in outcomes of patients.
